# Brazilian Road Traffic Fatalities: A Spatial and Environmental Analysis

**DOI:** 10.1371/journal.pone.0087244

**Published:** 2014-01-30

**Authors:** Luciano de Andrade, João Ricardo Nickenig Vissoci, Clarissa Garcia Rodrigues, Karen Finato, Elias Carvalho, Ricardo Pietrobon, Eniuce Menezes de Souza, Oscar Kenji Nihei, Catherine Lynch, Maria Dalva de Barros Carvalho

**Affiliations:** 1 Department of Nursing, State University of the West of Parana, Foz do Iguaçu, Parana, Brazil; 2 Department of Surgery, Duke University Health System, Durham, North Carolina, United States of America; 3 Duke Global Health Institute, Duke University, Durham, North Carolina, United States of America; 4 Department of Medicine, Faculdade Inga, Maringa, Brazil; 5 Instituto de Cardiologia do RS - Fundação Universitaria de Cardiologia, Porto Alegre, RS, Brazil; 6 Graduate Program in Informatics (PPGIA), PUC-PR, Curitiba, Parana, Brazil; 7 Department of Health Sciences, State University of Maringa, Maringa, Parana, Brazil; INDEPTH Network, Ghana

## Abstract

**Background:**

Road traffic injuries (RTI) are a major public health epidemic killing thousands of people daily. Low and middle-income countries, such as Brazil, have the highest annual rates of road traffic fatalities. In order to improve road safety, this study mapped road traffic fatalities on a Brazilian highway to determine the main environmental factors affecting road traffic fatalities.

**Methods and Findings:**

Four techniques were utilized to identify and analyze RTI hotspots. We used spatial analysis by points by applying kernel density estimator, and wavelet analysis to identify the main hot regions. Additionally, built environment analysis, and principal component analysis were conducted to verify patterns contributing to crash occurrence in the hotspots. Between 2007 and 2009, 379 crashes were notified, with 466 fatalities on BR277. Higher incidence of crashes occurred on sections of highway with double lanes (ratio 2∶1). The hotspot analysis demonstrated that both the eastern and western regions had higher incidences of crashes when compared to the central region. Through the built environment analysis, we have identified five different patterns, demonstrating that specific environmental characteristics are associated with different types of fatal crashes. Patterns 2 and 4 are constituted mainly by predominantly urban characteristics and have frequent fatal pedestrian crashes. Patterns 1, 3 and 5 display mainly rural characteristics and have higher prevalence of vehicular collisions. In the built environment analysis, the variables length of road in urban area, limited lighting, double lanes roadways, and less auxiliary lanes were associated with a higher incidence of fatal crashes.

**Conclusions:**

By combining different techniques of analyses, we have identified numerous hotspots and environmental characteristics, which governmental or regulatory agencies could make use to plan strategies to reduce RTI and support life-saving policies.

## Introduction

With economic growth, especially in low and middle-income countries, more vehicles are on the roads making daily transportation more complex and dangerous [1]. Road traffic injuries (RTIs) constitute a major public health challenge killing thousands of people prematurely every day, representing the leading cause of death for young people, which have increased by 46% over the last decade [1]–[2]. It is estimated that about 1.2 million people are killed in road crashes annually, and another 50 million people are injured. Additionally, it is expected that these numbers will increase by about 65% over the next 20 years [2]–[3].

RTIs lead to a considerable financial cost, particularly to developing economies. Low and middle-income countries have the highest annual road traffic fatality rates, accounting for 80% of road traffic deaths [1]. Indeed, RTIs are estimated to cost low and middle-income countries between 1–2% of their gross national product, estimated at over 100 billion dollars a year [1]. In Brazil alone, 43,908 people were killed in road traffic crashes in 2010, representing 3.86% of the deaths in the country [4].

Additionally, there is a substantial under-reporting of RTIs which limits our understanding of the range and types of injuries sustained in road crashes. This underreporting restricts the knowledge required to guide safety policies and delineate prevention strategies; therefore, further research on the incidence, types of crashes and detailed circumstances and contributing factors that lead to crashes are required [5]. Innovative analysis can identify injury hotspots and assess their built environments for dangerous characteristics, allowing for the creation of strategies to reduce RTIs and support life-saving policies [6].

In this context, many studies have used hotspots analysis [7]–[10], however this type of evaluation alone is not enough to support the creation of strategies for crash prevention. Many environmental factors appear to be associated and may have an amplifying effect on crash risk; therefore, a possible solution might be the use of environmental analysis together with hotspot analysis. These mixed analyses will provide a comprehensive understanding about the environment in which road traffic crashes occur. To date, there has been a limited exploration of these two analyses jointly in this context.

Thus, this study aims to map and spatially analyze road traffic fatalities locations, and determine the main environmental factors affecting road traffic fatalities on a Brazilian highway. We provide accurate local data analysis on road traffic fatalities, attempting to fit an existing gap of this type of data in the literature [11]–[13]. The results of this study will be used to inform resource allocation priorities and to guide interventions to address road traffic fatalities.

## Methods

### Institutional Review Board and Study Design

This study was approved by the Institutional Review Board of the State University of West of Parana (*Universidade Estadual do Oeste do Paraná* - UNIOESTE), in Brazil, under the registration number 316/2010. This is a descriptive, cross-sectional study of data obtained from the Brazilian Federal Highway Police Database on all road traffic crashes with fatalities that occurred on a Brazilian highway (BR 277), between 2007 and 2009.

### Highway Description

**Figure 1:**
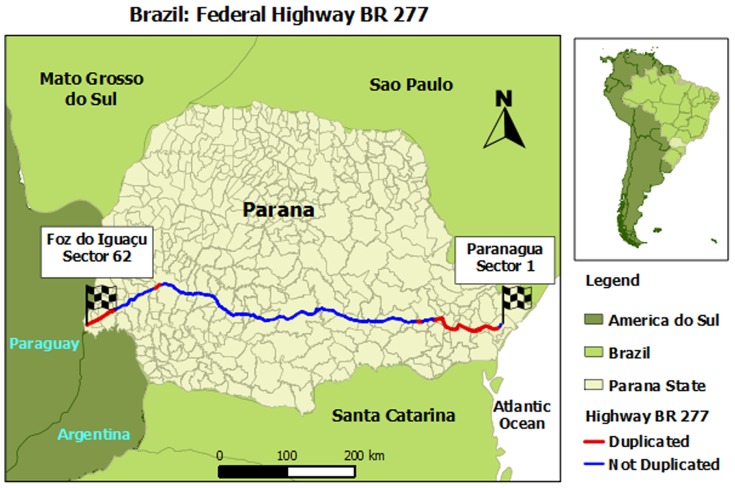
BR-277 Federal Highway, State of Parana, Brazil. Sources: Institute of Land and Cartography Geosciences website, 2010 [17] and Brazilian Institute of Geography and Statistics, 2010 [18].

This study analyzed the BR 277 highway located in the state of Parana, southern Brazil. This highway is 732.19 kilometers long, (548.46 km single and 183.73 km of double lane highway), traversing from east to west, from the city of Paranagua on the Atlantic Ocean Coast, through the state capital Curitiba and then directly to Foz do Iguaçu, on the border of Brazil with Argentina and Paraguay (Figure 1). This highway is divided into 62 sectors, beginning with Paranagua (sector 1) in the east and ending in Foz do Iguaçu (sector 62) in the west (Ministry of Transport - DENIT) [14]. For analysis purposes, only sectors 1 through 60 were included; sectors 61 and 62 were excluded since the Customs Officials of Brazil/Paraguay maintain records of road traffic incidents for these regions not the Federal Highway Police.

### Variables

Data were obtained from the Brazilian Federal Highway Police Database. We extracted variables related to fatal crashes events such as crash location, crash type, time of day, day of week, month and weather conditions. Crashes types included: pedestrian injuries, vehicular tipping or rollover, vehicular collision with bicycle, collision with fixed or mobile object, vehicular head-on/side impact, rear ended collisions, transverse collision (T-Bone), shedding of load, fall from a motorcycle/bicycle, or loss of control of the vehicle. The crashes were separated into two groups based on type of victim involvement: 1) Fatal pedestrian crashes, 2) Fatal vehicular crashes.

For the built environment evaluation, the inclusion criteria were characteristics of the environment created or modified by human interaction that might interfere in human health [15], enabling the proposal of solutions and improvements [16]. In order to collect the data, two of the researchers (LA, KF) crossed the entire BR 277 road collecting references of built environment variables and registering them with pictures. Fifteen environment variables were evaluated for the entire route of highway, which are described in the results section of this manuscript.

### Data Analysis

#### Spatial analysis

Spatial analysis was used to geographically specify the locations where the crashes occurred, and to assess specific patterns of distribution through map visualization. The georeferenced cartographic database of the state of Parana is freely available online in SHP format (shapefile) in the Institute of Land and Cartography Geosciences website [17] and Brazilian Institute of Geography and Statistics [18].

The analysis was conducted using a point process framework in order to identify sectors with the highest frequency of traffic crashes throughout BR 277. By understanding the spatial distribution of these points (crashes) in the geographic space, it is possible to evaluate observed patterns, determining if the data shows random characteristics, if it is deployed in clusters or if the points are evenly distributed [19]. The incidence of patterns was determined by spatial analysis by utilizing the open source software Quantum GIS (QGIS) version 1.9.0-Master [20] and a Kernel intensity estimator. This estimator establishes a two-dimensional function of events, forming a surface whose value is proportional to the intensity of samples per unit of area, so-called “hotspots” [20]. This function performs a count of all points within a region of influence, weighting them by the distance of each point to the location of interest.

Kernel intensity estimator uses first-order effects considered global or large-scale, corresponding to variations in the average value of the process space [18]. There are two basic parameters used in this function, the first is radius of influence (τ ≥0) which defines the neighborhood of the point to be interpolated and controls the smoothing of the surface generated, and the second is the function estimation with smoothing properties [18].

#### Wavelet analysis

A Wavelet analysis was used to complement the Kernel exploratory analysis. The analysis decomposes the series of number of deaths in each BR 277 highway sector into multi scales, producing a hotspot indicator by sector. This hotspot indicator is obtained by the statistically significant wavelet coefficients that represent the variance/energy explained in each sector. In this application, the analysis is performed in the finest scale, which carries the energy/variance due to the largest variations or the peaks of crashes (hotspots). The Autocorrelation Function (ACF) and/or the Augmented Dickey-Fuller (ADF) were used to verify if a series is stationary (not depend on time). As the series were non-stationary, the non-decimated wavelet analysis (NDWA) was performed [21]. Thus, effects that are hidden in time domain are revealed in time/frequency multi scale analysis. The wavelet basis used in the NDWA was Daubechies with 4 vanishing moments [22] and the implementation was carried out in R package [23].

This methodology allowed us to demonstrate that fatal crash hotspots are occurring repeatedly during consecutive years with the same behavior, not necessarily to the same value. In this sense, we built three geographic series of fatal crashes for each year. Correlating these smoother scales, we can check if certain events, in this case, the occurrence of accidents, behave the same way over the years. The Pearson correlation test was used to verify positive correlations between consecutive years as wavelet coefficients are uncorrelated and normally distributed.

#### Built environment analysis

After identifying hotspot locations, we performed further built environment analysis of these locations. Studies have shown that there are associations between the built environment and road traffic crashes involving pedestrian, cyclists, motorists on rural roads, highways and dense urban areas [24]–[25]. Environmental factors, such as curves, the poor weather conditions, inadequate lighting and signaling, and slippery road conditions are also correlated with a higher probability of occurrence of accidents, because drivers, cyclists and pedestrians may be more susceptible to accidents in these environments and driving conditions [26].

Initially, exploratory graphical analysis and descriptive statistics were used to evaluate the data. Univariate analysis was performed to compare each of the built environment variables between sectors of high and low risk of fatal crashes (defined with GIS and wavelet analysis). For group comparison, we used Mann-Whitney U test, and for variable association we used chi-square or Fisher’s exact test, when applicable.

To investigate the built environment’s impact on fatal crashes we applied a principal component analysis (PCA) to reduce the variables into similar variance components [27]. The number of components to be tested in the model was determined by Eigen-values (numbers higher than 1.00), screen plot analysis, communalities and factor interpretability (model with a theoretical rationale). PCA models were tested with promax rotation and the cutoff point of 0.40 was established for factor loadings.

All the built environment variables were entered in the PCA to evaluate the amount of components that explained the conjoint variance. Initial analysis with Eigen-values and scree-plot showed a possibility of three components, so models with one to four components were created and tested based on communalities (i.e. amount of variance from the observed variable that is explained by the model), factor loadings (i.e. amount of contribution of that observed variable to explain the component) and the theoretical rationale behind the components. The hypothesis with three components was the best solution so we chose the following built environment components: Urban Characteristics (UC), Road Surface (RS) and Road Configuration (RC). Urban Characteristics was constituted by observed variables that were mainly found on highway those transverse urban locations. Higher values indicate larger extension of the road inside urban area, presence of lighting, slower speed limit and less footbridges. The amount of road (in kilometers) inside urban area was defined through the classification found in the Brazilian Federal Highway Police Database.

Road Surface was configured by observed variables related to the static characteristics of surface of the road (higher values indicate higher length (in kilometers) of double lane roads, fewer presence of auxiliary lanes to single lane roads and more central unevenness). Auxiliary lanes are lanes that are added to one side of the single lane road, in areas of slow traffic to facilitate the flow. Road configuration was related to the dynamics of the road structure (higher values indicate more curves/km, more intersections/km and more slopes).

With the variables determined by PCA, we developed regression models to evaluate the impact of built environment components (independent variables) on fatal crashes (outcome variables). Due to the small number of observations (sectors) in the sample we opted to test three models with different groups of fatal crashes (vehicle collisions, pedestrian related crashes and road surfaces). Models were analyzed through regression diagnostics to evaluate their adequacy. Since the sectors were all of different lengths, all models were controlled for the possible impact of sector length on fatal crashes.

However, one of the main characteristics of BR 277 highway is its high heterogeneity among the sectors. Even inside each sector differences in built environment characteristics were observed. Therefore, we separated the sectors into clusters according to the environment components defined through PCA. The clustering method applied was the K-means. Each cluster characterized a pattern of build environment component indices. All analyses were performed in R language [23].

## Results

### Prevalence and Descriptive Characteristics of the Fatal Traffic Crashes

Between 2007 and 2009 there were a total of 379 crashes in all 60 sectors of the BR 277 highway. Some crashes had more than one fatality; therefore the total number of deaths was 466. One fatality was excluded due to a lack of data, thus 465 fatalities were analyzed. Fatalities were predominantly male (353 deaths, 75.9%), with an average of 117 deaths/year. The two age groups with the greatest occurrence of fatalities were from 31 to 50 years with 220 deaths (47.2%), and from 20–30 years with 119 (25.5%) of victims.

On weekends there were more fatalities than on weekdays with Saturday having the highest incidence (73 fatalities, 19.2%), followed by Sunday (70 fatalities, 18.4%). The month with the highest incidence of crashes was July with 42 fatalities (11.0%), however, when assessed the number of deaths due to crashes divided by the months, there were no significant differences.

Dividing hours of the day into 4 groups of 6 hours (06∶00 until 12∶00, 12∶00 until 18∶00, 18∶00 until midnight, and midnight until 06∶00) we observed that most fatal crashes occurred between 18∶00 and midnight (172, 45.3%), followed by midnight until 06∶00 (77, 20.3%). With respect to weather conditions, among the six climate modalities mentioned (clear skies, rain, fog, cloudy, sun and wind), clear skies were present for most of the fatal crashes (240, 51.6%), followed by cloudy weather (93, 20.0%).

The average number of fatalities per year on double lane versus single lane sections of BR 277 was 62±16 and 93±15, respectively. The fatalities per kilometer of single versus double lane road found an increased number of fatalities on double lane roads (2∶1 ratio).

In the eastern region of BR 277, a total of 48 pedestrian fatalities occurred likely due to population growth and urban development. The majority of this portion of highway has various sectors with appropriate conditions for road crossings and pedestrian crossings. The initial 5 km of highway in this region had 117 (25.2%) of the total fatalities during the study period. This high proportion was likely due to the single lane road, no shoulder and a large number of trucks entering into the Port of Paranagua, which is a main exporter of agricultural products from Brazil.

BR 277’s central region consists of 364 km of highway, mostly a single lane highway that traverses rural areas and smaller cities, and is separated from urban traffic. The total number of fatal crashes for this section in the period studied was 115 (24.73%). The most common type of crash was frontal impact collision (40), likely due to the lack of double lane roads for passing traffic and limited pedestrian road involvement. The second most common type of crash in this region was pedestrian related (29). This was likely due to the proximity of an indigenous population reserve juxtaposed to the highway increasing the pedestrian density along the roadway.

**Figure 2:**
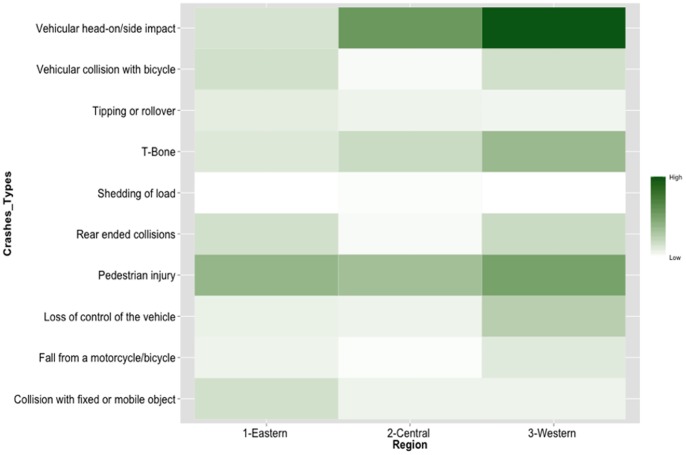
Heatmap - Number of fatalities by crash types in each region on highway BR-277.

Finally, the western region of BR 277 connects the city of Guaraniaçu and Foz do Iguaçu. This 210.3 km section of highway has 60.1 km of double and 150.2 km of single lane roadway. This region had a total of 233 (50.1%) of the fatal crashes during this study period. The main type of crashes in this region included front end collisions (77) followed by pedestrian injuries and transverse collisions (44, 32). Figure 2 demonstrates a heatmap comparing the number of fatalities by crash type in BR-277, during the studied period.

### Spatial Analysis and Wavelet Analysis

**Figure 3:**
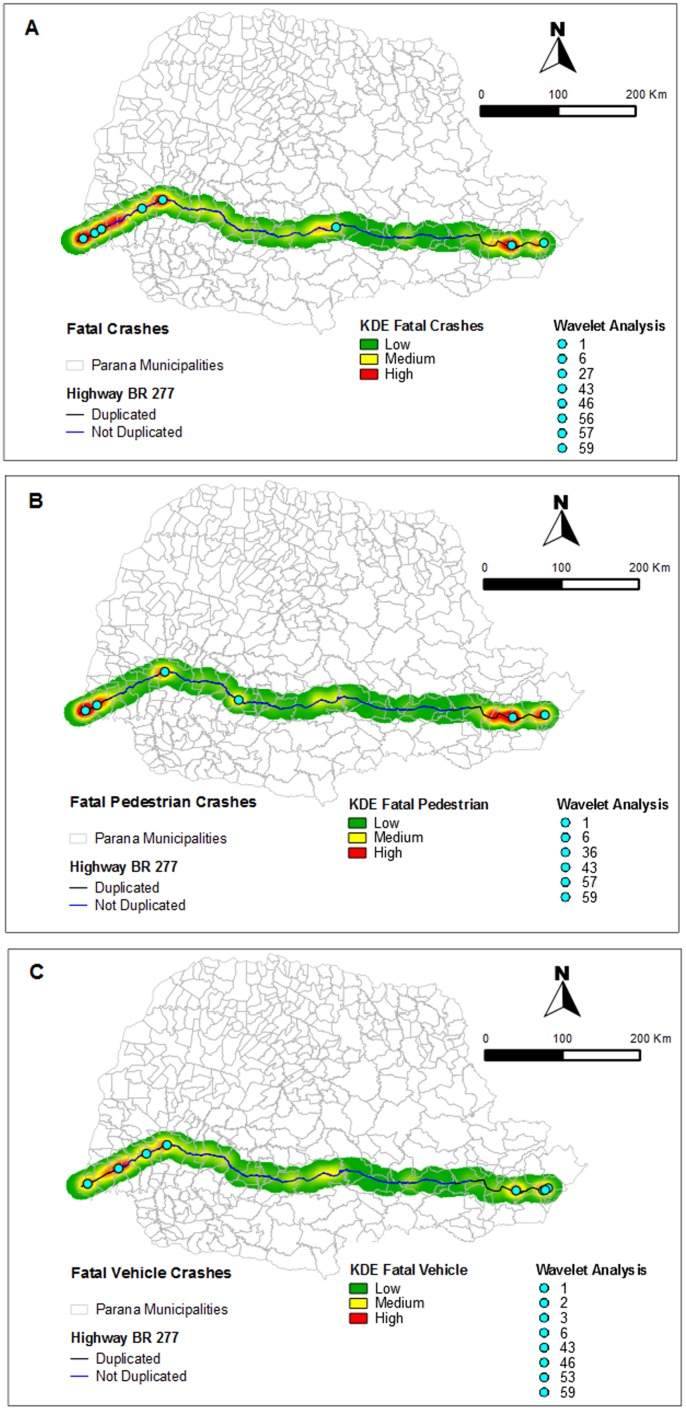
Kernel density and wavelet analysis hotspots. **3A)** All Fatal Crashes. **3B)** Fatal Pedestrian Crashes. **3C)** Fatal Vehicular Crashes.

The Map of Kernel (Figure 3A, 3B and 3C) showed a higher density (red) in eastern and western regions, with the lower density in the central region. In order to identify the sectors in which crash occurrences are statistically significant, the crash incidents between 2007 and 2009 were decomposed by NDWA. The wavelet coefficients are independent and normally distributed. Thus, in the spectrum (when squared), they follow chi-square distribution with 1 degree of freedom. From the confidence interval, the sectors with statistically significant (p<0.001) total crashes were 1, 6, 27, 43,46, 56,57,59 (Figure 3A), the sectors with statistically significant Fatal Pedestrian Crashes were 1, 6, 36,43, 57 and 59 (Figure 3B) and finally Fatal Vehicular Crashes were 1, 2, 3, 6, 43, 46, 53 and 59 (Figure 3C).

We can verify that the results the wavelet analysis and the kernel density have concordant results in the identification of hotspots. But, as discussed, it is as important to identify hotspots of crashes, as it is to show they are consistently hotspots during consecutive years.

The Pearson correlation test applied to fatal pedestrian crashes in 2007 and 2008 was 0.92 (p<0.0001, 95% CI 0.88, 1.00) and in 2008 and 2009 was 0.99 (p<0.0001, 95% CI 0.98, 1.00), suggesting very positive correlations. For the fatal vehicle crashes, correlations were 0.98 (p<0.0001, 95% CI 0.97, 1.00) and 0.90 (p<0.0001, 95% CI 0.86, 1.00), respectively. In this case, the occurrence of crashes, behaved the same way over consecutive years.

### Built Environment Analysis

From all the built environment variables (Table 1) length of road in urban area, auxiliary lanes and curve rate were able to differentiate sectors with high to low risk of fatal crashes in both pedestrian and vehicular fatal crashes. Sectors with high incidence of fatal pedestrian crashes were associated with higher length of road in urban area, limited lighting, more double lane roads, lower frequency of auxiliary lanes, and the presence of a traffic light. While larger road in urban area, few auxiliary lanes to single lane and smaller curve rate were associated with higher risk of fatal crashes with vehicles.

**Table 1:** Built environment variables evaluated for the entire route of BR 277.

Environment variables		Pedestrian (Mean (SD) or N (%))		Vehicles (Mean (SD) or N (%))	
		High Risk	Low Risk	High Risk	Low Risk
Length of road in UA (Km)		5.00 (3.71)	0.00**	2.40 (3.56)	0.00**
Intersections (rate by Km)		0.36 (0.30)	0.23 (0.27)**	0.40 (0.40)	0.20 (0.23)**
Curves (rate by Km)		0.16 (0.24)	0.39 (0.30)**	0.19 (0.29)	0.43 (0.28)**
Slopes	No	8 (73)	35 (71)	22 (76)	21 (68)
	Yes	3 (27)	14 (29)	7 (24)	10 (32)
Traffic lights	No	9 (82)	48 (98)	27 (93)	30 (97)
	Yes	2 (18)	1 (2)*	2 (7)	1 (3)
Police stations	No	7 (64)	39 (80)	22 (76)	24 (77)
	Yes	4 (36)	10 (20)	7 (24)	7 (23)
Bus stops	No	4 (36)	33 (67)	16 (55)	21 (68)
	Yes	7 (64)	16 (33)	13 (45)	10 (32)
Side Unevenness	No	9 (82)	45 (92)	25 (86)	29 (93)
	Yes	2 (18)	4 (8)	4 (14)	2 (7)
Speedbumps	No	8 (73)	43 (88)	25 (86)	26 (84)
	Yes	3 (27)	6 (12)	4 (14)	5 (16)
Lighting	No	4 (36)	36 (73)	17 (59)	23 (74)
	Yes	7 (64)	13 (27)**	12 (41)	8 (26)
Speed limit	<80	6 (54)	17 (35)	14 (48)	9 (29)
	110	5 (46)	32 (65)	15 (52)	22 (71)
Footbridge	Yes	7 (64)	41 (84)	25 (86)	23 (74)
	No	4 (36)	8 (16)	4 (14)	8 (26)
Number of Lanes	D	9 (82)	9 (18)	12 (41)	6 (19)
	S	2 (18)	40 (82)*	17 (59)	25 (81)
Auxiliary Lanes	No	10 (91)	26 (53)	23 (79)	13 (42)
	Yes	1 (9)	23 (47)**	6 (21)	18 (58)*
Central Unevenness	No	8 (73)	44 (90)	24 (83)	28 (90)
	Yes	3 (27)	5 (10)	5 (17)	3 (10)

SD: standard deviation; *P<0.05; **P<0.01; D = Double Lanes; S = Single Lane; UA = Urban Area.

**Figure 4:**
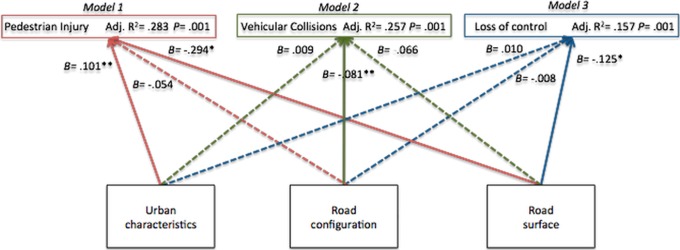
Built environment components’ impact on fatal crashes divided into Pedestrian Injuries (Model 1), Vehicular Collisions (Model 2) and Loss of control of the vehicle (Model 3).

Built environment components were able to explain the variance of pedestrian related crashes in 28%, vehicular collisions in 25% and loss of control of vehicle in 15%. Vehicular collisions, pedestrian related crashes and road surfaces (Model 1) were positively predicted by the urban perimeter component. Collisions were only significantly negatively impacted by road configuration (Model 2) while the average occurrence of loss of control of the vehicle diminished with larger road surface component (Model 3) (Figure 4).

**Figure 5:**
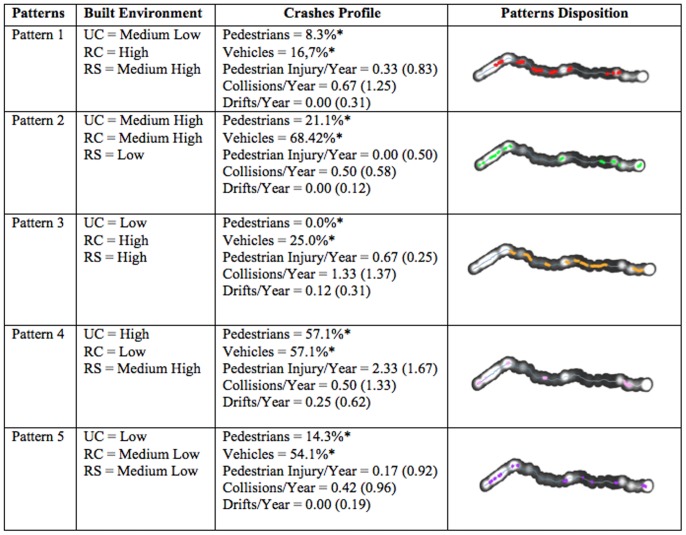
Geographic distribution and characteristics of the five built environmental patterns according to categories with the sectors of high and low risk of fatal crashes.

These results demonstrate that specific environmental characteristics are associated with different types of fatal crashes, indicating sector specific interventions. Combining the built environment components to cluster the sectors from BR 277, we obtained 5 different patterns (Figure 5).

Patterns 2 and 4 are the ones constituted mainly by large urban characteristics and therefore, have frequent fatal pedestrian crashes. Although the two patterns differ in relation to RS and RC, what may affect the profile of fatal crashes. Pattern 2, for instance shows more duplicated areas and an urban perimeter more structured towards safety. On the other hand, patterns 1, 3 and 5 display mainly rural characteristics and have higher prevalence of vehicular collisions. Patterns 1 and 3 are similar but pattern 1 has a high rate of intersections while pattern 3 has more slopes and curves. Pattern 5 is anomalous with low rates in all built environment components (double lanes, in rural areas, low rates of curves, intersections and slopes); however, pattern 5 has a high speed limit and increased traffic.

## Discussion

To our knowledge, this was the first study of road traffic crash hotspot locations with environmental factor analysis on a Brazilian highway. The overall 379 crashes yielded 466 fatalities on BR 277 between the years 2007 and 2009. A higher incidence of crashes occurred on sections of highway with double lanes. Additionally, the hotspot analysis demonstrated that both the eastern and western regions had higher incidences of crashes when compared to the central region. In the built environment analysis, variables significantly associated with a higher incidence of fatal crashes were length of road in urban area, limited lighting, double lane roadways, and less auxiliary lanes.

The literature mirrors our data from BR 277 demonstrating that front-end collisions are the leading type of crash followed by pedestrian injuries [28]. Similarly, our data correlate with the literature finding that favorable climate, night time hours (18∶00–00∶00) during weekends and the 31–50 age male groups are factors often associated with fatal crashes, and these findings are even more predominant in urban settings.

While a high incidence of fatal crashes during favorable climate seems counter intuitive, it is postulated that favorable climatic conditions could decrease driver’s attention and potentially increase their speed and the risk of crashes and fatal injuries. In our study, the most prevalent victims were males during their ‘economically productive’ ages, between 31 and 50 years. Similar data are observed in the literature [29]. Recent data from Brazil suggest that 81.5% of road traffic crashes victims are males who are primarily injured during pedestrian crashes followed by car, and motorcycle crashes [30]. This male predominance may be because of their risk taking behavior, which has been previously described in the literature to be more prevalent than among women [31].

Several studies show that there is a larger number of crashes in urban stretches of highways, due to the heavy traffic of vehicles, several intersections for local traffic, inadequate traffic engineering, and inappropriate behavior of pedestrians and drivers [28]. The addition of a second lane in each direction creating double lane sections has been described as a possible solution to reduce crashes [32]–[34]. Paradoxically, this study found that double lane sections of road were more dangerous than single lane roads. A possible explanation for this finding may be the higher density of pedestrian and vehicular traffic in the urban locations near the double lane roads. It is important to note that most double lane road sections of BR-277 are when the highway traverses cities. Additionally, a second lane can potentially allow for an increased speed of traffic, which has been found to be associated with increased risk of fatal RTI. Also, there is a large number of tourists in the east and west regions of the BR-277 highway, whose are usually not familiar with the road regulations or conditions, which may lead to a higher incidence of crashes.

Similarly, since the State of Parana is one of the largest producers of grains in Brazil there is a predominance of trucks and agricultural vehicles on the highways, which are difficult to maneuver and may limit visibility in the lanes. Ultimately, recklessness and excessive speed are risk factors on all stretches of the highway but have a higher chance of causing fatal injury or crash in urban environments due to the higher density of pedestrians.

As expected, previous study of RTI in Brazil between the years 1998 and 2010 found higher mortality rates of pedestrian injuries than of other types of injury [35]. In our data we found that pedestrian injuries were most prevalent in urban environments. Such environments had a high density of pedestrians and vehicular traffic, increased speed and driver recklessness, in addition to more complex traffic patterns, which is supported by the literature [36]–[39].

Fatal RTI hotspot identified through kernel density analysis highlighted two high hotspots in the eastern and western region (hot regions) of BR-277. We utilized both kernel density and wavelet analysis over a period of years to show the persistence of these hotspots in consecutive years. By identifying highway road traffic injury hotspots with a combination of different traditional or more sophisticated methods of analysis plays a key role in the prevention and reduction strategies in the high-density areas of crashes [40]–[43].

In the eastern region hotspot, a 5 km road section, it was observed 16 fatal crashes in three years given an average of 5.3 deaths per year. This section was single lane, no shoulder, with intense flow of vehicles, especially trucks in direction to the Port of Paranagua, largest exporter of agricultural products from Brazil. In the western region, we evidenced the sectors 56 and 57, with 16 fatal crashes to each sector averaging 5.3 deaths annually. These sectors are doubled lane, with sinuous soft inside the urban environment, did not have pedestrian walkways, only two pedestrian tunnels, unevenness of median, uphill and downhill in unaccented in both directions. However, the fatalities were mainly from pedestrian injuries and loss of control of vehicle.

Another hotspot in the western region occurred in the urban area Foz do Iguaçu (section 59) near the end of the BR-277. This 15.6km section of highway had a total of 35 fatalities averaging 11.67 fatalities annually. In this section of highway, there is a high density of vehicular traffic especially in the late afternoon. In addition there are complex traffic patterns, with only a walkway for pedestrians and with roadside bus stop in both directions.

Among the various hotspots identified along the highway, a hotspot drew attention by the most common type of crash; pedestrian injury. This hostspot was the sector 37 located in the central region of the highway; this section with 22.2 km of extension had 13 deaths and an annual average of four deaths. Given the proximity of a Native Indian reservation juxtaposed to the highway, there is a popular belief that Indians utilize the highway to access nearby drinking establishments and return to the reservation walking on the side of highway. Further analysis into these actions is warranted and alternative methods of transportation, pedestrian safety interventions and population education are encouraged by our findings.

It is observed that many changes in road infrastructure were made over the past decades, but have not reduced the number of deaths and injuries on the roads, whether in urban and rural areas [44]. However the built environment has a direct influence on the occurrence of accidents, as well as the specific behaviors that cause them. It is necessary to better understand how the patterns of the environment identified in this study influence the traffic of vehicles, cyclists and pedestrians, as well as seasonal influences related to transporting crops and tourist movements at both ends of the state to prevent and reduce accidents and fatalities on the highway BR 277.

This study has some limitations. The data obtained from the Brazilian Federal Highway Police Database only included the road traffic crashes that had death on scene. Our data did not include any of the road traffic crashes where the patients died on the way to the hospital or during the hospital stay. Therefore, this analysis represents only the most severe road traffic crashes that caused instantaneous death. Further research is needed not only on the immediately fatal crashes but also on all road traffic crashes to better describe the burden of all road traffic injuries in this region of Brazil. Similarly, further analysis is warranted including hospital based data on road traffic crash, the volume of traffic to be able to calculate rates of road traffic crashes, and not just incidences, and further crashes details to describe crash demographics, such as passenger rather than driver fatalities.

In conclusion, we combined in this project four different techniques for identifying and analyzing road traffic injury hotspots on the BR 277 highway in Parana, Brazil. By combining geographic information systems, wavelet analysis, built environment analysis and principal component analysis we have found 5 patterns of hotspots, 2 for pedestrian and 3 for vehicular fatal crashes along BR 277. Finally, we have been able to augment traditional geographic information systems outputs to have a more comprehensive understanding of road traffic injury hotspots for which we have created tailored unique and innovative interventions for road traffic injury in Brazil.
